# The Editor's Letter-Box

**Published:** 1917-01-27

**Authors:** 


### THE EDITOR'S LETTER-BOX.

### Churches and Charity Funds.

*To the Editor of* THE HOSPITAL.

SIR,—There is something very pitiable in the announcement to a congregation in church that "The collection will be given to the hospitals after the payment of church expenses." It certainly creates a very unpleasant feeling in the hearer; there must be "something wrong" when a congregation habitually contributes to the collection on such a scale as to make such an announcement necessary. The fact is people have never taken to heart that almsgiving is as much a Christian duty as prayer, and must also be regular and not spasmodic. The apostolic injunction directing the weekly contribution has hardly ever been driven home, and in times like the present collections suffer and the churchwardens are filled with apprehensions. Money is given "grudgingly and of necessity," for they dare not stand wholly aside, and a percentage is exacted.

A parallel case is the Divine injunction as to fasting, which though insisted on in the Prayer-book is practically ignored, and at the present time the State is invoked to enforce a weekly "meatless day," which, if the clergy had done their duty, would already exist for a very large proportion of the population. Again, few clergy have the faculty of making a lucid financial statement (and this disability is not confined to the clergy), so that the people may learn exactly what it costs "to run" a church. If the habitual attendants once grasped the actual necessary expenses of their place of worship they would doubtless take a larger share in helping the churchwardens meet their financial responsibilities, and such notices would automatically cease.—I am, Sir, yours faithfully,

PRESBYTER.

